# Motor Imagery Acquisition Paradigms: In the Search to Improve Classification Accuracy

**DOI:** 10.3390/s25196204

**Published:** 2025-10-07

**Authors:** David Reyes, Sebastian Sieghartsleitner, Humberto Loaiza, Christoph Guger

**Affiliations:** 1 School of Electrical and Electronics Engineering, University of Valle, Cali 760032, Colombia; humberto.loaiza@correounivalle.edu.co; 2 g.tec Medical Engineering, 4521 Schiedlberg, Austria; 3 Institute of Computational Perception, Johannes Kepler University, 4040 Linz, Austria

**Keywords:** Electroencephalogram (EEG), motor imagery accuracy, common spatial patterns, novel paradigms, brain computer interface (BCI)

## Abstract

In recent years, advances in medicine have been evident thanks to technological growth and interdisciplinary research, which has allowed the integration of knowledge, for example, of engineering into medical fields. This integration has generated developments and new methods that can be applied in alternative situations, highlighting, for example, aspects related to post-stroke therapies, Multiple Sclerosis (MS), or Spinal Cord Injury (SCI) treatments. One of the methods that has stood out and is gaining more acceptance every day is Brain–Computer Interfaces (BCIs), through the acquisition and processing of brain electrical activity, researchers, doctors, and scientists manage to transform this activity into control signals. In turn, there are several methods for operating a BCI, this work will focus on motor imagery (MI)-based BCI and three types of acquisition paradigms (traditional arrow, picture, and video), seeking to improve the accuracy in the classification of motor imagination tasks for naive subjects, which correspond to a MI task for both the left and the right hand. A pipeline and methodology were implemented using the CAR+CSP algorithm to extract the features and simple standard and widely used models such as LDA and SVM for classification. The methodology was tested with post-stroke (PS) subject data with BCI experience, obtaining 96.25% accuracy for the best performance, and with the novel paradigm proposed for the naive subjects, 97.5% was obtained. Several statistical tests were carried out in order to find differences between paradigms within the collected data. In conclusion, it was found that the classification accuracy could be improved by using different strategies in the acquisition stage.

## 1. Introduction

Recent research has shown that, worldwide, there are around 65 million people who have suffered some kind of Traumatic Brain Injury (TBI), which has led them in difficult situations where motor injuries can occur and, in the worst-case scenario, leading them to quadriplegia [1]. Among the most complex situations where people can survive with some kind of motor injury are stroke, Spinal Cord Injury (SCI), Amyotrophic Lateral Sclerosis (ALS), Multiple Sclerosis (MS), cerebral palsy, among others [2]. In Colombia, there are around 770,000 people in this kind of situation, according to the National Administrative Department of Statistics (DANE) [3]. To try to mitigate the effects of these matters, interdisciplinary research has been carried out where doctors, engineers, scientists, and researchers from different fields of expertise which work together to develop tools that allow these people to communicate, interact [2], restore some kind of mobility, etc. To this end, Brain–Computer Interfaces (BCIs) have played an important role, which, although it is an emerging technology, has proven to be a powerful tool when applied in aspects related to rehabilitation and functional restoration [4]. There are several methods that work with BCIs; however, this work will focus on those that involve motor imagery (MI) from EEG biosignals. This corresponds to motor tasks that are performed internally and mentally without their execution. MI-based BCIs have been used globally in rehabilitation therapies where patients can somehow, after a series of repetitions and training, improve their situation [5]. This type of BCI is commonly used to control, for example, a neuroprosthesis [6,7]. There are also works where they use this method in conjunction with Functional Electrical Stimulation (FES) for treatment of gait [8], for post-stroke therapies [9], and even in combination with robotics [10].

Although different works and research have been reported that indicate the effectiveness of MI-based BCIs in rehabilitation and functional restoration processes, likewise, the need has been seen to review aspects that involves more attention from BCI users, since the user’s attention, concentration and motivation plays a fundamental role in the processes of MI repetition and feedback to obtain better results in the rehabilitation therapy [5]. Regularly in BCI training stages, subjects can choose several strategies to carry out motor imagination; however, the system could provide good instructions on how to carry out this imagination. In some works, they recommend carrying out EEG signal acquisition paradigms of MI where those tasks are related to familiar actions that are or were executed daily [11]. This way, the user could have better performance with a BCI.

Different strategies have been considered to mitigate the low accuracy rate on the recognition of MI tasks for example advances in signal processing and machine learning methods [12]. Others focused on the feature extraction stage using e.g., the CSP algorithm in MI recognition [13] or even by using deep learning methods [14]. In response for seeking better classification accuracy this work proposes, three kinds of paradigms that were designed for the acquisition of motor imagination tasks through EEG biosignals: the first is the traditional method where an arrow cue as stimulus is used; the second is a picture of a hand used as cue so that users concentrate on their own hands; and the third is a video of how to perform the motor imagery action. In the context of motor rehabilitation, BCIs establish a bridge between users’ motor tasks and external devices, e.g., computers, an electrical wheel chair [15], exoskeletons [16], and prosthetic hands [17], among others. Such motor tasks encompass motor execution, motor attempt, or motor imagery (MI), with all of them leading to event-related desynchronization (ERD) and synchronization (ERS) over sensorimotor areas [18]. Therefore, these decreases (ERD) and increases (ERS) in oscillatory power are often utilized as control signals for MI-based BCIs. Additionally, ERD is associated with greater cortical activation [19]. The main objective is to identify those ERD from EEG recordings by using the strategies, previously mentioned, at the acquisition stage of the data to be processed. The rest of the article is organized as follows: Section 2 explains the materials, methods, and the experimental setup. Section 3 shows the results obtained for the different experiments, in Section 4, a discussion is presented, and, in Section 5, some conclusions about the work performed are provided.

## 2. Materials and Methods

**Figure 1:**
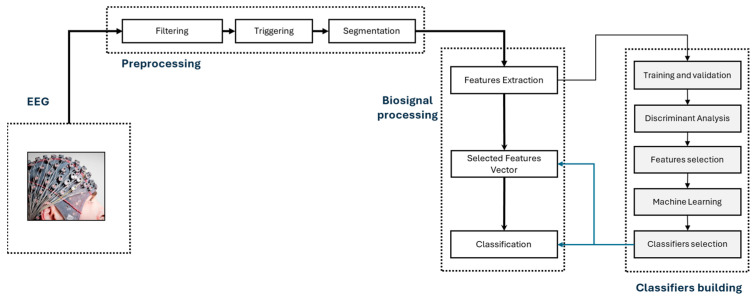
Schematic diagram of the framework followed by the proposed methodology.

This section will explain the materials and methods used for the acquisition, pre-processing, and processing of the biosignals obtained for the purposes of motor imagery tasks recognition. The guideline of the proposed methodology was followed by the framework shown in Figure 1.

The primary objective of this work is to improve the accuracy rate in the recognition of MI tasks from EEG signals by using different paradigms during the acquisition stage of the data. The next section will explain in detail the implementation made.

### 2.1. EEG Biosignals Acquisition

**Figure 2:**
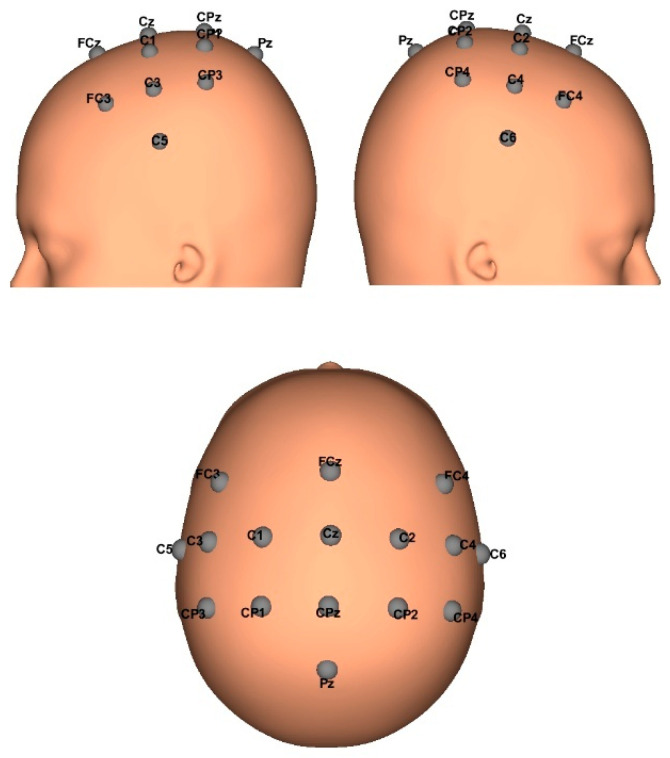
Distribution of the 16 EEG biosignal acquisition channels around the motor cortex.

Data was obtained with the approval of the Ethikkommission des Landes Oberösterreich in Austria (#D-42-17), Post-stroke EEG signals were obtained from three subjects with left and right hand involvement, and data from ten healthy subjects (aged 22–44 years) with no diagnosis of brain disease or body mobility. All healthy participants agreed to and signed informed consent and declared no experience with MI-BCIs or BCIs. A g.Nautilus PRO device, manufactured by the Austrian company g.tec medical engineering GmbH, with 16 acquisition channels, was used. EEG biosignals were sampled at a frequency of 250 Hz, conduction gel was applied to improve signal quality on each channel, and raw EEGs were obtained for further processing in MATLAB v.2023a. The EEG electrode positions were FC3, FCz, FC4, C5, C3, C1, Cz, C2, C4, C6, CP3, CP1, CPz, CP2, CP4, and Pz; according to the international 10/20 system, a reference electrode was placed on the right earlobe and a ground electrode at AFz. This electrode distribution allowed recording brain electrical activity focused on the motor cortex, which is where cortical activations occur when imagining or performing a motor movement. The following Figure 2 shows the distribution of the electrodes of the montage made for the acquisition of EEG signals.

#### Experimental Paradigms

The participants were prepared for the brain electrical activity recording using EEG, for which a shielded space was organized containing a 20-inch LED monitor with a resolution of 1920 × 1080 pixels and a refresh rate of 60 Hz. This monitor is connected to the computer which controls the start and end of each run. Initially, a black screen with a fixation cross is displayed, indicating to the user that data recording is about to begin. In this work three types of acquisition paradigms were used: (1) the traditional arrow paradigm, (2) a hand picture paradigm and (3) a hand video paradigm.

**Figure 3:**
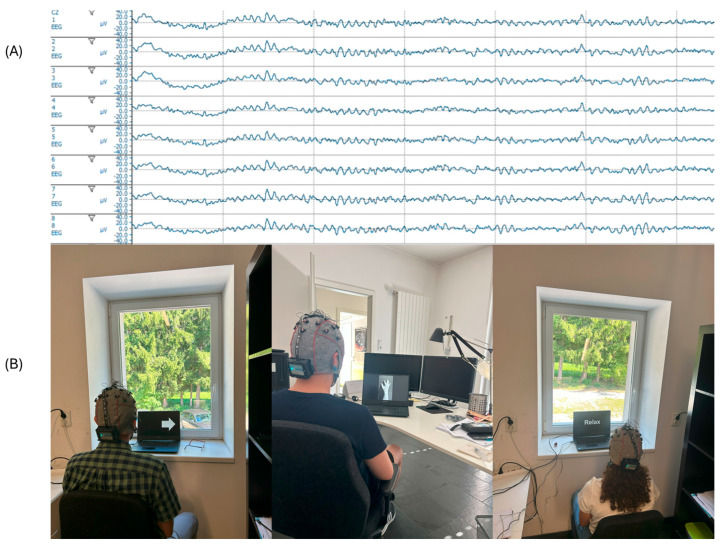
(**A**) First 8 of 16 EEG biosignals channels captured using the g.Nautilus PRO distributed around the motor cortex. (**B**) Experimental setup for data recording.

To acquire the EEG data, it was suggested that each user would not make strong and unnecessary movements; they would be relaxed and in the same way concentrated on the motor imagination task, for which they were initially explained what type of action they should imagine. Each user performed two types of motor imagination tasks: imagining moving the left hand and the right hand. These actions were shown randomly during the acquisition process, obtaining 40 trials per class for a total of 80 trials per subject for each paradigm, for a total of 240 trials per subject. The order of each paradigm was shown randomly for each subject in each session. Figure 3 shows the biosignals acquired (A) and the experimental setup (B).

**Figure 4:**
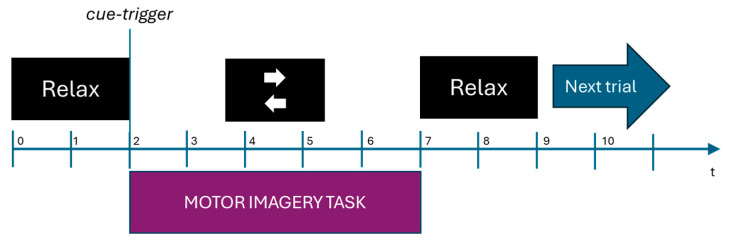
Traditional arrow paradigm timing for a single trial in seconds used for EEG signal recordings.

For the naive subjects, the traditional arrow paradigm consisted of showing an arrow pointing left or right, indicating which side should be imagined; this paradigm is commonly used [20,21]. As shown in Figure 4, on a black screen, a fixation cross followed by a “Relax” message is shown at the beginning of each trial, indicating to the subject that the motor imagination task recording is going to start. Then an arrow cue, after 2 s, is shown pointing to which side MI should be performed (left or right). Subjects had 5 s to perform the MI task. Finally, a notice is shown for each subject to relax and prepare for the next trial.

**Figure 5:**
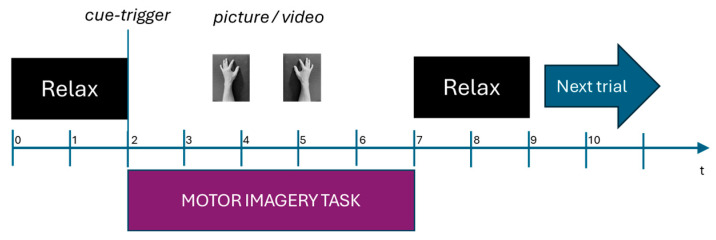
Hand picture paradigm and grasping hand video paradigm timing for a single trial in seconds used for EEG signal recordings.

The other and novel paradigms, the hand picture paradigm and hand video paradigm, consisted of showing the user which hand they should imagine for the case of the picture, and for the video, the action they should or could imagine. Similarly, a fixation cross is shown on a black screen, indicating that the experiment is going to start, followed by a picture or video cue indicating the hand that must be imagined. The MI task is performed for 5 s for each subject; after this, a “Relax” message is displayed, and the subject is prepared for the next trial. Figure 5 shows the timing of the paradigms proposed in this work.

The implemented methodology was also tested with data from experienced BCI users after a stroke. Data was captured using the recoveriX [22] rehabilitation system from g.tec medical engineering, in 3 real-time runs for each user. During the acquisition and classifier calibration process, feedback was generated using Functional Electrical Stimulation (FES) and a 3D avatar. Thus, it was possible to validate that the proposed methodology was capable of identifying brain activity related to motor imagery of the left and/or right hand. The recordings and capturing protocol were the same as those used in the experiment with healthy individuals, and present the same protocol as the BR41N.IO Hackathons [23]. Testing and post-processing of the obtained data were applied in the same way as with the inexperienced healthy subjects. Table 1 shows the description of the data for each post-stroke participant and their condition.

**Figure 6:**
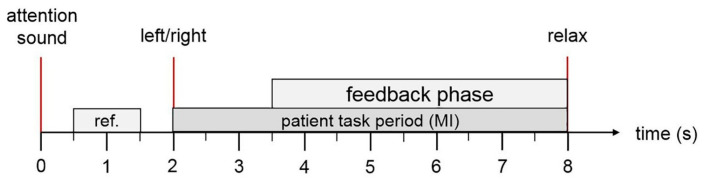
Paradigm protocol used to capture EEG biosignals for MI tasks. Adapted from [18].

Figure 6 shows the paradigm timing used for data capturing, where it is observed that after obtaining the indication cue to perform the motor imagination task, 1.5 s later, feedback is provided with the aspects mentioned previously.

### 2.2. EEG Biosignals Processing

After obtaining the raw EEG data, it was organized in MATLAB for further processing. Initially, a signal filtering process is carried out between 8 and 30 Hz, which, according to several works [11,24,25], is where electrical activation occurs during the execution and imagination of motor tasks. For this, a 4th-order Butterworth bandpass filter was designed, as well as a 3rd-order Notch type to filter the 50 and 60 Hz powerline. These filters have shown great results in several works [19,26], and their implementation does not require high computational resources. A Common Average Referencing (CAR) filter, Equation (1), was also applied; this kind of filter allows us to remove typical or common activity from the EEG biosignals and preserves the inactive activity of each individual signal at a specific electrode. This reference method was used to improve the signal-to-noise ratio (SNR) caused by artifacts in EEG signals. With this method, removing the average across all electrodes could produce cleaner signals [2]. CAR helps minimize uncorrelated sources of the biosignal and noise through averaging, while eliminating sources of noise common to all sites. Therefore, a common average reference more closely approximates the theoretical differential recording ideal [27].

(1) $$\tilde{x}\left(t\right)=x_{i}\left(t\right)-\frac{1}{N}\sum_{j=1}^{N}x_{j}\left(t\right)$$

where

(2) $$x_{i}\left(t\right):\;\mathrm{raw}\;\mathrm{EEG}\;\mathrm{signal}:\;\mathrm{channeEEG}\;i\;\mathrm{in}\;\mathrm{time}\;t$$

(3) $$\frac{1}{N}\sum_{j=1}^{N}x_{j}\left(t\right):\;\mathrm{mean}\;\mathrm{value}\;\mathrm{of}\;\mathrm{all}\;\mathrm{channels}\;\mathrm{in}\;\mathrm{time}\;t$$

(4) $$\tilde{x}\left(t\right):\;\mathrm{signal}\;\mathrm{of}\;\mathrm{channelEEG}\;i\;\mathrm{after}\;\mathrm{CAR}$$

Subsequently, the trials were organized according to each class, subject and paradigm, considering the starting trigger of each MI task. For this, the starting point was taken 2 s before the MI cue trigger is shown and 2 s after finishing the MI task, so the total length of the trial is 9 s. Figure 6 shows the organization of the trials structure.

**Figure 7:**
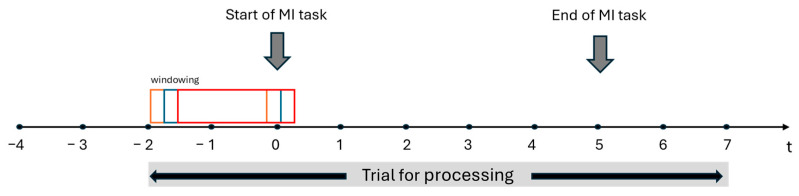
Trial structure, organization, and windowing for signal processing for each class, paradigm, and subject.

Once the trials were organized, signals were obtained for each class, which were then taken to a windowing process of T = 2 s and T = 3 s with steps of 0.2 s (see Figure 7), in order to evaluate the accuracy of the classifier throughout the entire run. Then, the Common Spatial Pattern (CSP) was applied to the data for transforming it into a new matrix with minimal variance of one class and maximal variance of the other [13,28]. This method is based on the simultaneous diagonalization of two covariance matrices whose decomposition of the EEG data leads to a new time series that allows two classes to be discriminated.

Given 16 channels of EEG data for each trial, left and right X, the CSP method returns a projection matrix W of size 16 × 16. The decomposition of the trials would then be given by Z = WX. According to several works [28,29,30] by selecting the first 2 and the last 2 rows of the matrix W, an optimal CSP method can be obtained to reduce the dimensionality of the new EEG data. Finally, the feature vector is organized by calculating the variance, taking into account the prior windowing T and log transforming the data:

(5) $$f=\log\left(\frac{{VAR}_{r}}{\sum_{r=1}^{4}{VAR}_{r}}\right)\;$$

For analysis and classification, two different types of classifiers were implemented: a Linear Discriminant Analysis (LDA), a Support Vector Machine (SVM) with two kernels, linear and 3rd-order polynomial. LDA classifiers are commonly used in several works [31,32,33,34], and also SVM for the classification of motor imagery data [11,35,36]. The accuracy was estimated via 10-fold cross-validation, which means that the data was partitioned into 10 subsets: for training and others for validation and testing. The accuracy was calculated for each paradigm and subject every 0.2 s steps for all trials, and the session accuracy was the maximum value after calculating the average of the 10 cross-validation repetitions.

## 3. Results

To illustrate the results obtained, different figures are shown with the accuracy for each subject, evaluating two window sizes, T = 2 s and T = 3 s. The size T = 1 s was discarded due to poor performance in terms of accuracy. Likewise, images are presented that refer to the Event Related Desynchronization and Synchronization (ERD/ERS) for the best and the worst performance during the tests, along with their CSP filter distributions. In this work, the healthy subjects were new to motor imagination tasks and had never even used a BCI, which is why the accuracy threshold for good performance was established to be greater than 60%. There are works that have reported good performance from this threshold [11]. The results are shown: first, the ones obtained for the healthy participants, followed by the post-stroke subjects.

**Figure 8:**
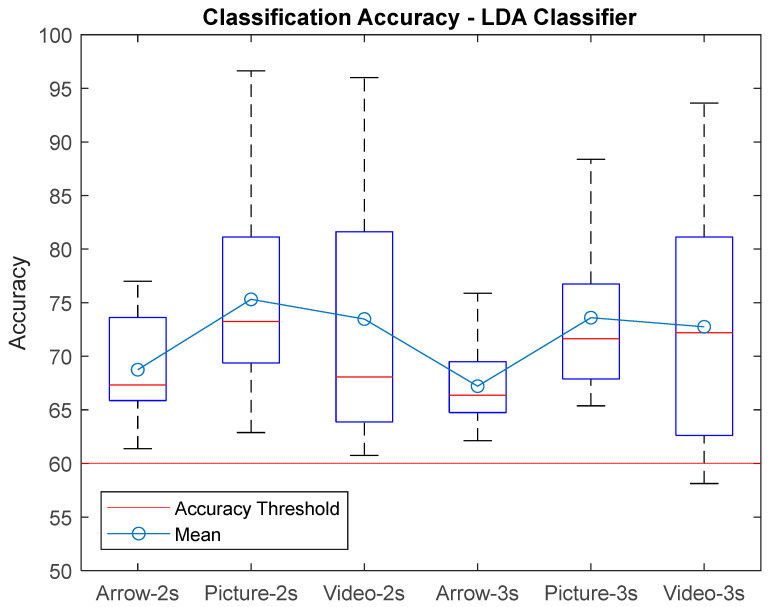
Classification accuracy for the LDA classifier using window size T = 2 s, T = 3 s for each paradigm presented to gather the EEG biosignals from MI task.

Figure 8 presents the accuracy boxplot for each paradigm for the selected window sizes corresponding to the LDA Classifier. It can be seen that the majority of subjects obtained an accuracy percentage greater than 60% and also that the best classification rates were for the proposed paradigms. The maximum accuracy was for the picture paradigm classification, reaching 96.62% with a window size of T = 2 s, and the lowest was for the video paradigm, reaching 58% with a window size of T = 3 s.

**Figure 9:**
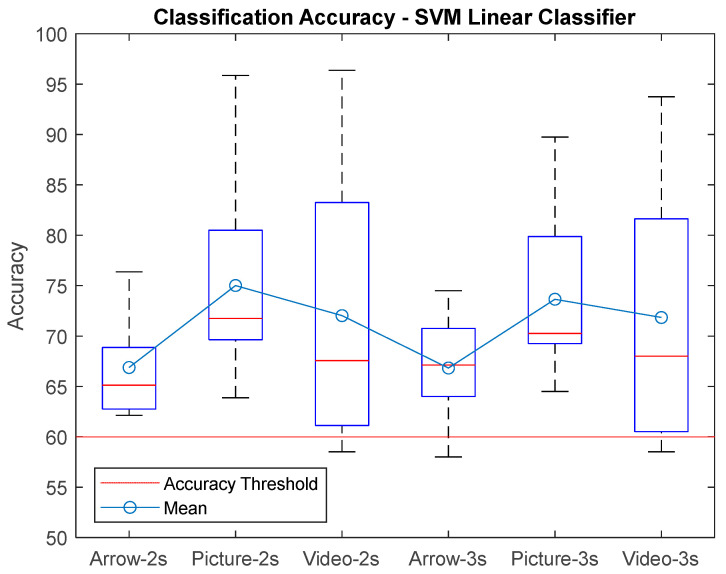
Classification accuracy for the SVM-Lin classifier using window size T = 2s, T = 3 s for each paradigm presented to gather the EEG biosignals from MI task.

**Figure 10:**
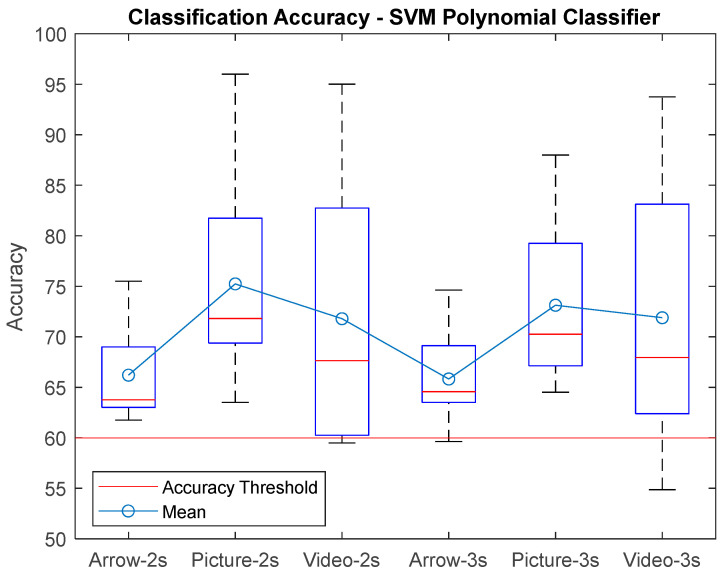
Classification accuracy for the SVMPoly classifier using window size T = 2 s, T = 3 s for each paradigm presented to gather the EEG biosignals from MI task.

Figure 9 shows the results boxplot for the SVM-Lin classifier, where the improvements in accuracy can be seen compared to the traditional arrow paradigm. It is important to mention that all subjects had an accuracy above 60% for the hand picture paradigm, which indicates that the performance was good for all naive subjects for this paradigm. The best performances were for window size T = 2 s. Figure 10 shows the results for the SVM-Poly classifier hand video paradigm, where it is observed that there is also an increase in the accuracy if compared with the arrow paradigm for some subjects; however, the lowest classification accuracy was performed with this classifier for 54.8% for window T = 3 s.

**Figure 11:**
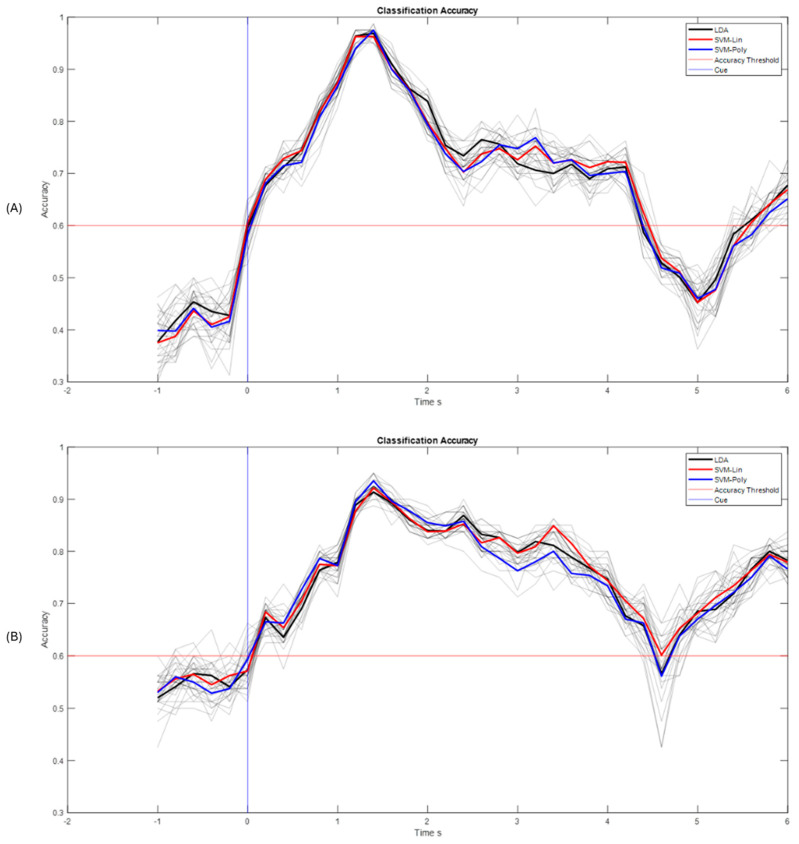
Classification accuracy best performance subjects (**A**) S10 for hand picture paradigm and (**B**) S7 for hand video paradigm, window size T = 2 s.

With these results it can be concluded that the best performance was reached within a time processing window of T = 2 s, and only two subjects did not reach the threshold accuracy of S2 and S5 with this time window size. Likewise, in Figure 11 one can see the best accuracies that correspond to subjects S7 and S10 for the MI_Video and MI_Picture paradigms for each implemented classifier using a time window size of T = 2 s. It is highlighted that subject S10 obtained an accuracy above 90% for both paradigms.

**Figure 12:**
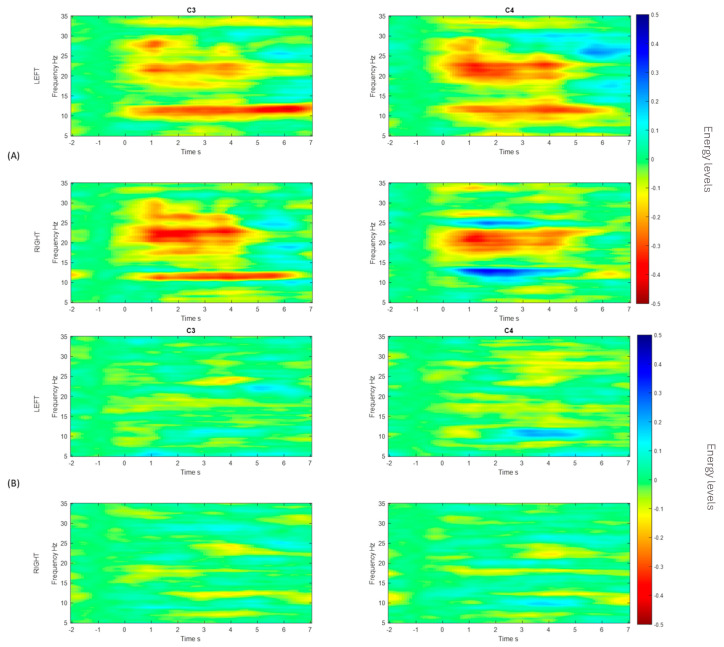
ERD/ERS maps for best (**A**) and worst (**B**) performance for channels C3 and C4 of the EEG biosignals related to left- and right-hand MI tasks.

Figure 12 shows the ERD/ERS obtained for channels C3 and C4, respectively. These channels show electrical activation when a motor imagination task occurs: when the action corresponds to a right-hand motor imagery task, the energy was lower at C3, and vice versa for the C4, when the energy from the left-hand motor imagery task was lower [11]. In motor imagery paradigms, the ERD is observed contralateral to the imagined limb, whereas the ERS can appear ipsilaterally or during the post-stimulus resting phase [37]. These patterns allow us not only to understand the neurophysiological mechanisms underlying motor imagery, but also to construct discriminative features useful for EEG MI-based BCI systems [38]. The ERD/ERS of the best and worst performers in relation to the accuracy of the classifier are shown (see Figure 12), where a change in the energy levels (A) is observed in comparison to the worst performer (B).

Similarly, using the g.BSanalyze software(g.tec, Upper Austria, Austria), it was possible to calculate and graph the CSP filters; the first two and last two CSP filters are shown. These patterns or filters reflect the spatial distribution of MI-based neuronal activity and are essential for feature extraction, allowing for the distinction between different motor imagination classes, such as left and right hand MI. In the intraindividual analysis, the distribution of patterns is different, suggesting interindividual variability in cortical activations during MI tasks [12]. CSP filters maximize the signal variance for one class and minimize it for the other, resulting in characteristic spatial patterns. When these filters are projected onto the scalp as topographic maps, the areas with the greatest weight reflect the cortical regions that exhibit significant differences in EEG oscillation power. In the case of motor imagery, these maps typically show differentiated activations over the contralateral sensorimotor regions (mainly around areas C3 and C4 of the 10–20 system). For example, during right-hand imagery, it is common to observe greater modulation in electrodes close to C3 (left hemisphere), while, for the left hand, the opposite occurs around C4 (right hemisphere). It is important to note that CSP maps do not directly represent neuronal activity, but rather the spatial distribution of the filter weights that best separate the classes. Therefore, they should be interpreted as indicators of the scalp regions that contribute most to the discrimination of mental states associated with the task [28].

**Figure 13:**
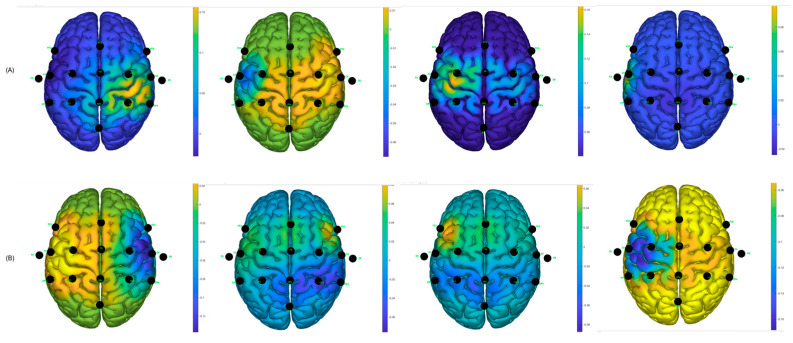
CSP filter distribution for S10, (**A**) Traditional arrow paradigm. (**B**) Proposed, hand picture paradigm.

It can be observed, in Figure 13, that there is a variability in the spatial distribution of the CSP components in the comparison of the best S10 performance for the traditional paradigm A, with the proposed picture paradigm B. In Figure 12B, it can be observed that there is a contralateral activation between the first and the last filter, which can lead to obtaining a good percentage of success in motor imagination task recognition.

**Figure 14:**
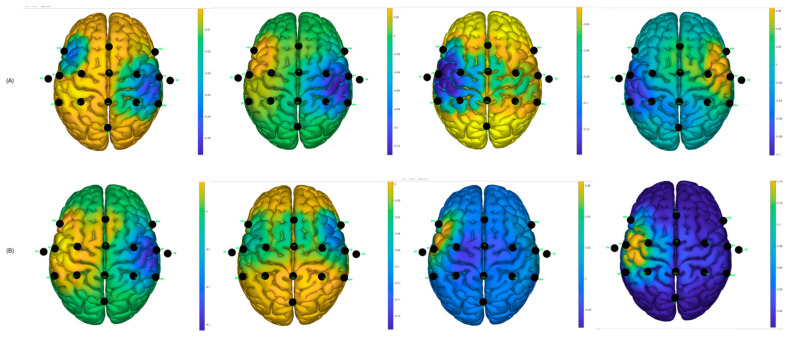
CSP filter distribution for S7, (**A**) Traditional arrow paradigm. (**B**) Proposed, hand video paradigm.

For participant S7, the same analysis was performed and it was found, according to Figure 14, that the filters are distributed better for the hand video paradigm, in which the subject obtained the best success performance, compared to the distribution of the traditional arrow paradigm.

**Figure 15:**
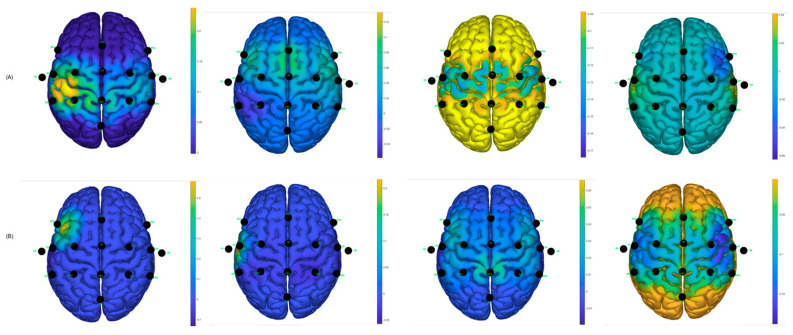
CSP filter distribution. (**A**) Traditional arrow paradigm for S4. (**B**) Traditional arrow paradigm for S8.

Two distributions are shown randomly for two participants who obtained low success rates in motor imagery recognition tasks; see Figure 15. It can be observed that the distributions are not as discriminating as for the other cases; this likely affects the performance of the classifiers and creates greater confusion between the predicted output data and the actual output data.

Table 2 shows the results obtained for each classifier, window size, subject, and paradigm. It is observed that the best accuracy percentages are achieved for the windows of size T = 2 s and that, in comparison with the results obtained with the traditional arrow method, the proposed MI_Picture and MI_Video paradigms present better accuracy percentages for subject S10, who obtained the greatest improvement, an increase of 20.5%.

**Figure 16:**
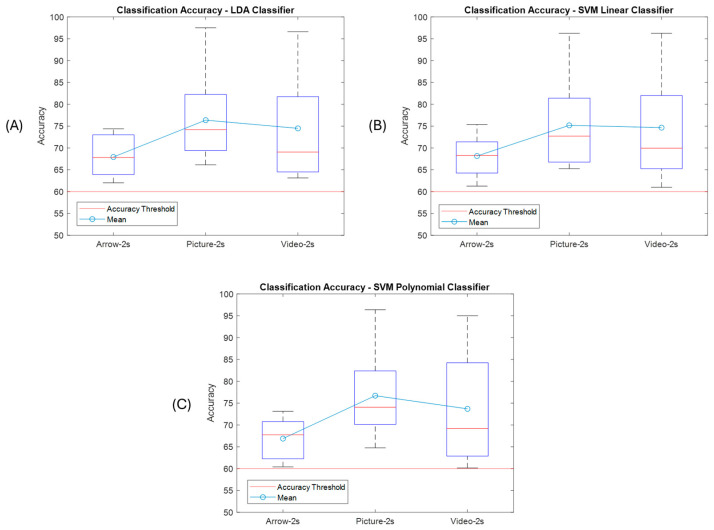
Subject accuracy for each classifier and each acquisition paradigm implemented, with CAR filtering. (**A**) Arrow paradigm. (**B**) Hand picture paradigm. (**C**) Hand video paradigm.

Finally, another test was carried out using CAR filtering to check the classification accuracy performance. Figure 16 shows the boxplots obtained after CAR. In Table 3, which summarizes the results obtained after applying the CAR filter, an interesting improvement can be observed compared to the previous results, where some classifiers could not reach the accuracy threshold; in this case, all of them exceeded it. The best performance was obtained with an accuracy of 97.5% for both participant S10 for the proposed hand picture paradigm and for participant S7 for the video paradigm, whose improvement for this occasion was 23.13% and 22.38%, respectively, compared to the traditional paradigm. It was also found that an improvement in the accuracy was obtained for the hand video paradigm, in general, for all users.

A Wilcoxon signed rank test, ANOVA, and Bonferroni-corrected *t*-tests were implemented to show the classification accuracy differences with a significance threshold set to α = 0.05. Table 4 shows the results for the *p*-value (values *p* < 0.05 are in bold) obtained after the test by comparing the traditional arrow paradigm accuracy with the proposed paradigms. Table 5 shows the *p*-values obtained after applying the CAR filter and for a window size of T = 2 s, since it had the best classification accuracy performance during the previous tests.

Observing the results obtained, it can be seen that there are some important aspects for the comparison between paradigms within the data processed, for example, after obtaining the Bonferroni-corrected *p*-value, there is a significance difference (*p* < 0.05) between arrow vs. picture and arrow vs. video for SVM classification and for both window sizes T. Better results are obtained after CAR filtering in which all classifiers showed significant differences after Bonferroni-corrected tests for both comparisons.

### Post-Stroke Data

**Figure 17:**
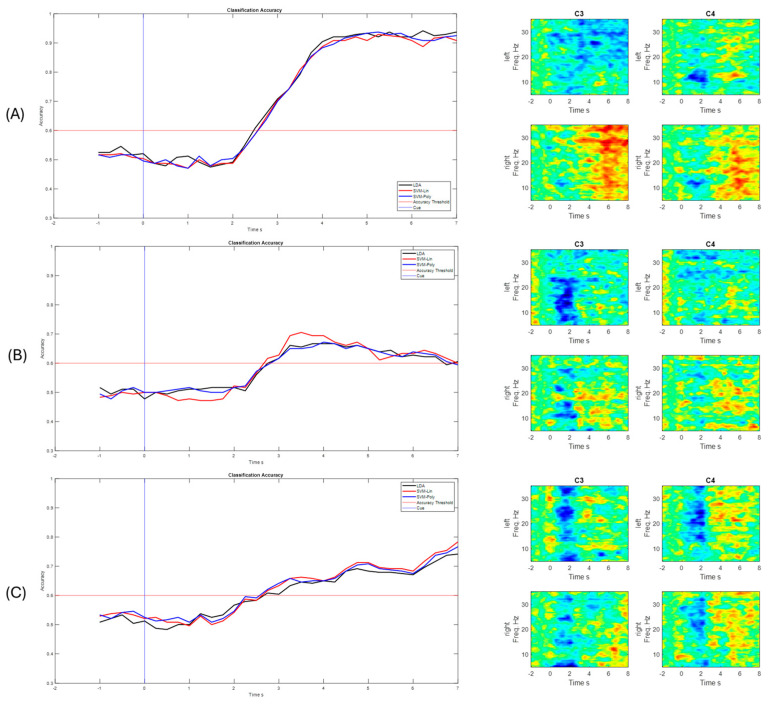
Classification performance for each post-stroke user and for each implemented classifier. (**A**) S1—Run 3. (**B**) S2—Run 3. (**C**) S3—Run 3.

For the post-stroke data, a total of 40 imagination tasks were performed by each hand and both hands during each of the 3 runs, for a total of 120 MI tasks for each of both upper limbs. The same steps as the proposed methodology were performed, implemented, and tested, with the results shown in the following graphs. The data were processed with a processing window of t = 2 s, as it obtained the best performance in the previous tests and validations. In the first run, the classifier was trained, and the CSP filters were calculated. Run 2 and Run 3 were used to test the classifier obtained in the previous run. Figure 17 shows the classification performance for the last run for each subject and their ERD/ERS.

**Figure 18:**
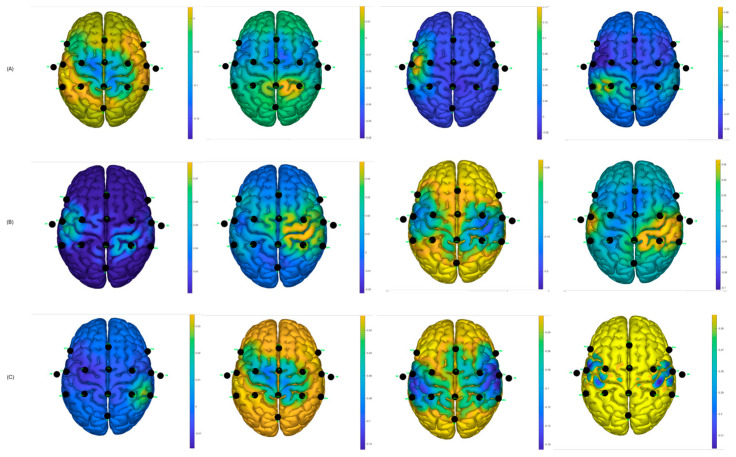
CSP filter distribution for post-stroke subjects: (**A**) Subject 1—Left Hand affected. (**B**) Subject 2—Left Hand affected. (**C**) Subject 3—Right Hand affected.

Figure 18, shows the CSP filter distribution for each subject for the classifier calibration run. This calibration stage is one of the most important aspects since testing runs would perform according to the results obtained during the classifier calibration. It can be observed that, for example, in Figure 18A there is a discriminative distribution that could lead to good performance in the classification rate, and there are high values around the left hemisphere of the brain for filters 3 and 4, which could indicate that it was easier for the subject to perform MI for the non-paretic hand. This could be found for the other subjects as well.

**Figure 19:**
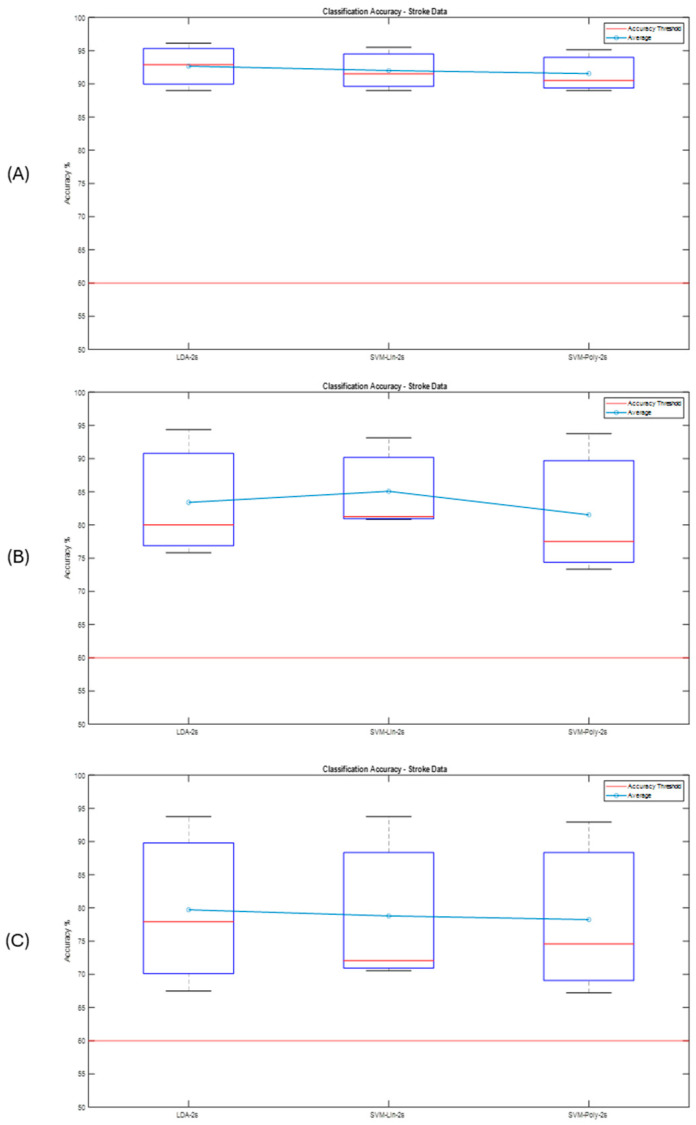
Subject classification accuracy for each classifier implemented with a t = 2 s window. (**A**) Run 1—Calibration. (**B**) Run 2—Testing. (**C**) Run 3—Testing.

In Figure 19, it can be observed that all accuracy percentages exceeded the defined accuracy threshold, with above 60%. The best-performing user was S1, with a maximum correct answer of 95.42% with the LDA classifier, for Run 3. It is important to note that the three classifiers performed well in identifying motor imagery tasks. It can also be observed that for 3 subjects there is an ERD/ERS activity related to the motor imagination tasks, and that the accuracy improves after getting to the feedback phase, which FES can have a strong role in contributing to ERD generation [39].

## 4. Discussion

**Figure 20:**
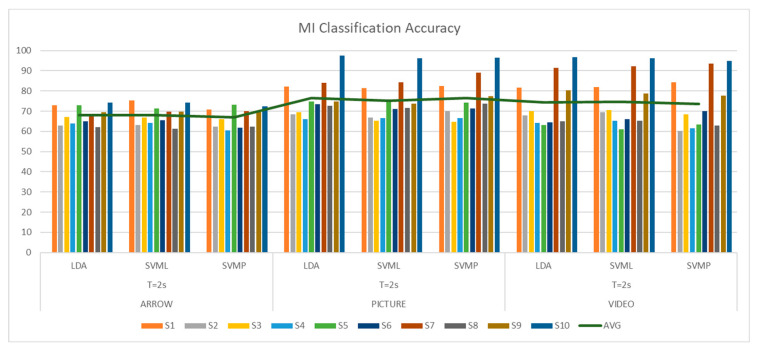
Motor imagery performance for each healthy subject for the best classification accuracy reached for processing time window T = 2 s.

When analyzing the results obtained, there is a significant difference in the data gathered in relation to the accuracy obtained with the classifiers implemented for the proposed paradigms. The paradigm corresponding to the hand picture stands out, which obtained a significant difference, *p* < 0.05, for all classifiers and both processing time window sizes. Processing time window size classification accuracy performed better for the T = 2 s (see Figure 20), which also leads to a fast response in the processing pipeline. Healthy subjects stated that they feel more comfortable with the paradigms that show how to perform the motor imagery action, or which hand they have to imagine. Works [40] have shown that cortical brain activation can be induced by several factors, such as intended, observed, and/or imagined, the action to perform. This is an interesting aspect that is congruent with the results obtained. It is important to mention that BCIs are first and algorithm-dependent, but, on the other hand, subject-dependent as well. That is probably why there are some subjects that performed better than others.

The best classification accuracy was 97.5% for the hand picture paradigm obtained from subject S10; in fact, S10 performed the best or at least one of the best for all the paradigms and classifiers. Subjects S1, S7, and S10 stated that they are sporty and healthy humans, which could lead to performing better in MI tasks compared to the others, who were mostly workers and students. For example, during the analysis of the CSP filters, the distribution was better and more discriminative for these subjects than for the ones that had lower performance (see Figure 12, Figure 13 and Figure 14). This could also induce the presence of ERD/ERS during the motor imagery tasks within those subjects who had better performances. It is important to mention that action observation, repetitive actions, and performing familiar actions generate changes in power around mu rhythm [41], which is the main frequency band that is used in this work.

Some subjects reported that the video paradigm was confusing for them, but others found it easier to imagine the motor imagery action. This could be explained by the activation of the mirror neuron system introduced by [42], where it might play a fundamental role in both action understanding and in the capacity to learn by imitation. Within the arrow paradigm, subjects performed the MI task by using several strategies, and all of them felt more comfortable with the picture one. Some works have reported that through an observation of the action or an indication of it, brain activity has been evidenced in the premotor cortex, in the supplementary motor area, and in the primary somatosensory cortex [11]; this may be associated with the better performance obtained with the proposed paradigms compared to the traditional one.

For the post-stroke results, it was noted that the use of the FES and VR as feedback could generate a better motor imagery performance in each subject. The feedback phase plays an important role during rehabilitation processes that seek to restore functionality [19,26,43]. As for the BCI performance, when using feedback during the calibration stage, it improved the results during the training of the classifiers to be tested in the next runs. However, further studies with larger data are necessary to demonstrate, for example, the mirror neuron activation, and to obtain a more generalized classification.

**Figure 21:**
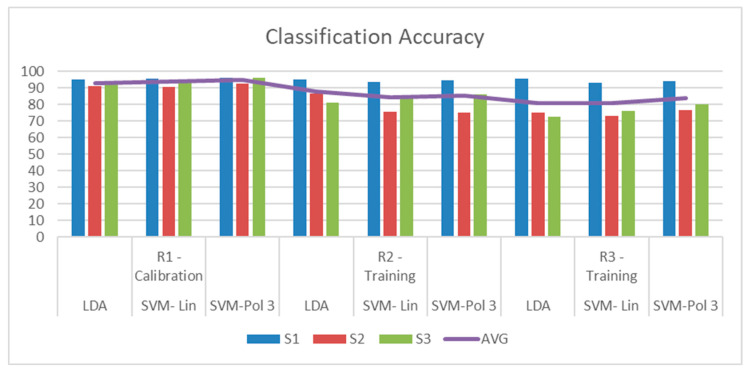
Post-stroke motor imagery classification accuracy for each run and classifier implemented.

With the results obtained from the best performances, and, in general, from the post-stroke data, something interesting was observed in the classification curve: after around ~1.5 s, it can be seen how the classification accuracy improves. This is because after that period of time, feedback through the FES and 3D Avatar was applied, which could help the user to better perform the motor imagination tasks. Similarly, Event-Related Desynchronizations and Synchronizations (ERDs/ERSs) can be observed when the MI tasks occur, which may indicate that it is actually the motor imagination that is recognized by the proposed methodology. The best classification performances were obtained with the LDA classifier, since it obtained the highest percentage of success in Run 3, where data from Run 2 was also processed and classified. However, the other classifiers are within the range with similar percentages of success. Figure 21 shows the summary of the results obtained.

## 5. Conclusions

In this work, three paradigms were tested for motor imagery-based BCIs using a hand picture and a hand video in comparison to the traditional arrow. The experiment demonstrated that the subjects who participated in the study improved the classification accuracy significantly, *p* < 0.05. It was also demonstrated that by using familiar actions, the MI task could be performed better. The paradigms proposed could induce motor imagery (MI)-related ERD, as well as action observation (AO) ERD, because both actions generate cortical activity; in fact, experiments that involve AO+MI, such as the ones in this work, could enhance that cortical activity [44]. Similar strategies to [11] were performed by subjects to perform MI tasks. It is very important to take into account that BCI is mostly subject-dependent and this study showed that with naive BCI users, it can obtain good performance by using different approaches and strategies in the acquisition paradigm; adding some feedback in this stage could also improve the results for further studies [19,45], as demonstrated in this work for the post-stroke data.

The results obtained in this work showed good performance in terms of the role within the results reported in the literature. The best performances (97.5%-S10 and 93.5%-S7) in this work are in the range of [11], where a Chinese character is presented in the paradigm stage. This work also presents better results than the best performance in this work [13], where they used visual robotic feedback in the paradigm, and the best accuracy is around 92%. This work surpasses those results of recent works, such as [12], where they obtained 95.24% in the recognition of the MI task.

For future works, an adaptive BCI framework could be implemented, and closed-loop paradigms show promising results for improving BCI performance in future implementations. The use of a window size T = 2 s, as well as the low computational costs, methods, and techniques used in this work, could be good for real-time response implementation. Using other approaches of BCI could be an opportunity for future work; invasive Electrocorticography (ECoG)-based BCI are demonstrating to have outstanding results, for example, in quadriplegic patients [46].

## Figures and Tables

**Table 1:** Description of the condition of post-stroke participants.

Participants	Condition
S1	Hand: Left side affected
S2	Hand: Left side affected
S3	Hand: Right side affected

**Table 2:** Motor imagery accuracy percentage for each subject, classifier, and paradigm, having window sizes of T = 2 s and T = 3 s.

MOTOR IMAGERY ACCURACY																		
PARADIGM	ARROW						PICTURE						VIDEO					
**WINDOW**	T = 2 s			T = 3 s			T = 2 s			T = 3 s			T = 2 s			T = 3 s		
**CLASSIFIER**	LDA	SVML	SVMP	LDA	SVML	SVMP	LDA	SVML	SVMP	LDA	SVML	SVMP	LDA	SVML	SVMP	LDA	SVML	SVMP
SUBJECT																		
S1	73,62	75,25	72,25	75,87	74,5	74,62	81,12	80,5	81,75	76,75	79,87	79,25	81,63	83,25	82,75	81,13	81,63	83,13
S2	75,87	62,75	64,25	63,12	65,12	63,5	62,88	63,87	66,87	65,38	64,5	64,5	65,5	63,5	59,5	62,62	63,5	62,25
S3	65,87	65,63	63	62,12	61,5	63,5	69,37	69,63	69,38	70,87	69,87	71,63	70,63	71,88	70,5	72,25	72,5	69,5
S4	66,75	64,62	63	64,75	64	63,75	67,87	66,75	63,5	65,63	66	66,37	63,88	62,62	60,25	59,63	59,88	54,87
S5	67,87	67,88	67,88	68	69,12	69,12	73,13	74,25	71,88	67,88	70,63	67,13	60,75	58,5	60,13	58,13	58,5	62,38
S6	64,62	63	61,75	65,37	64,12	60,5	73,75	71,37	71,37	71,63	71,88	70,38	63,88	60,5	66,63	63,88	60,5	62,62
S7	68,37	68,87	69	67,37	69,12	67,12	83,13	84,38	86,5	86,12	84,88	84,38	90,5	90,88	92,5	87	87,63	87,38
S8	61,38	62,13	62,13	65,37	58	59,62	71,88	72,13	71,75	71,62	69,25	70,12	60,75	61,12	62	72,12	61,12	66,38
S9	66,13	62,25	63,25	70,63	72	65,38	73,37	71,37	73,38	71,75	69,87	69,5	81,25	71,62	68,63	77,13	79,38	76,63
S10	77	76,37	75,5	69,5	70,75	71,12	96,62	95,87	96,38	88,38	89,75	88	96	96,38	95	93,62	93,75	93,75
**AVG** **± STD**	68,75 ± 5,09	66,88 ± 5,24	66,20 ± 4,75	67,21 ± 4,05	66,83 ± 5,12	65,83 ± 4,71	75,31 ± 9,52	75,01 ± 9,48	75,28 ± 9,91	73,60 ± 7,94	73,65 ± 8,34	73,13 ± 7,98	73,48 ± 12,93	72,02 ± 13,62	71,79 ± 13,51	72,75 ± 12	71,84 ± 12,98	71,89 ± 12,74

**Table 3:** Motor imagery accuracy percentages for each subject, classifier, and paradigm, having window size of T = 2 s after applying the CAR filter.

MOTOR IMAGERY ACCURACY									
**PARADIGM**	ARROW			PICTURE			VIDEO		
**WINDOW**	T = 2 s			T = 2 s			T = 2 s		
**CLASSIFIER**	LDA	SVML	SVMP	LDA	SVML	SVMP	LDA	SVML	SVMP
SUBJECT									
S1	73	75,37	70,75	82,25	81,38	82,38	81,75	82	84,25
S2	62,87	63,25	62,38	68,5	66,75	70,13	68	69,38	60,13
S3	67	66,75	66,13	69,38	65,25	64,75	70,12	70,5	68,38
S4	63,88	64,25	60,38	66,13	66,62	66,5	64,25	65,25	61,63
S5	73	71,38	73,13	74,88	74,88	74,37	63,13	61	63,38
S6	65	65,63	61,88	73,5	71	71,25	64,5	66	70
S7	68,63	69,75	70	84	84,25	89,13	91,38	92,13	93,5
S8	62	61,25	62,25	72,63	71,63	73,75	64,87	65,25	62,87
S9	69,5	69,75	69,38	74,88	73,75	77,5	80,25	78,75	77,75
S10	74,37	74,13	72,5	97,5	96,25	96,38	96,62	96,25	95
AVG ± STD	67,93 ± 4,50	68,15 ± 4,69	66,88 ± 4,84	76,37 ± 9,31	75,18 ± 9,65	76,61 ± 10,04	74,48 ± 12,24	74,65 ± 12,13	73,69 ± 13,20

**Table 4:** Summary of *p*-values obtained with the statistics test for the classification accuracy from the paradigms comparison: arrow vs. picture and arrow vs. video for both window sizes T = 2 s and T = 3 s.

CLASSIFIER	PARADIGMS	*p*_ANOVA	Wicoxon	*p*_Bonferroni-Corrected	*p*_ANOVA	Wilcoxon	*p*_Bonferroni-Corrected
LDA	Arrow vs. Picture	**0,0412**	**0,0488**	0,1235	**0,0201**	**0,0039**	0,0602
	Arrow vs. Video	0,2110	0,3750	0,6331	0,1315	0,1602	0,3946
SVM Linear	Arrow vs. Picture	**0,0016**	**0,0020**	**0,0048**	**0,0130**	**0,0195**	**0,0390**
	Arrow vs. Video	0,1398	0,3223	0,4195	0,1669	0,2324	0,5008
SVM Polynomial	Arrow vs. Picture	**0,0013**	**0,0020**	**0,0040**	**0,0059**	**0,0059**	**0,0177**
	Arrow vs. Video	0,1161	0,1602	0,3483	0,0971	0,1309	0,2912

**Table 5:** Summary of *p*-values obtained with the statistics test for the classification accuracy from the paradigms comparison: arrow vs. picture and arrow vs. video after applying the CAR filter for window size T = 2 s.

		T = 2 s		
CLASSIFIER	PARADIGMS	*p*_ANOVA	Wilcoxon	*p*_Bonferroni-corrected
LDA	Arrow vs. Picture	**0,0032**	**0,0020**	**0,0096**
	Arrow vs. Video	0,0699	0,0600	0,2096
SVM Linear	Arrow vs. Picture	**0,0104**	**0,0039**	**0,0312**
	Arrow vs. Video	0,0660	0,0400	0,1980
SVM Polynomial	Arrow vs. Picture	**0,0026**	**0,0059**	**0,0077**
	Arrow vs. Video	0,0737	0,0800	0,2212

## Data Availability

The datasets are available upon request to the authors. The main reason the dataset has to be requested is that participants’ data need to be treated according to current data protection laws and ethical guidelines. So, requests to access the datasets should be directed to D.R.: david.reyes@correounivalle.edu.co.
